# Triethyl-Borates as Surfactants to Stabilize Semiconductor Nanoplatelets in Polar Solvents and to Tune Their Optical Properties

**DOI:** 10.3389/fchem.2022.860781

**Published:** 2022-04-12

**Authors:** Yalei Deng, Xufeng Chen, Jing Liang, Yuanyuan Wang

**Affiliations:** State Key Laboratory of Coordination Chemistry, School of Chemistry and Chemical Engineering, Nanjing University, Nanjing, China

**Keywords:** triethyl-borate ligand, semiconductor nanoplatelets, ligand exchange, surface chemistry, optical properties

## Abstract

Stabilizing nanocrystals (NCs) with high fluorescence quantum efficiency in suitable solvents and tuning of their optical properties precisely are critical for designing and assembling optoelectrical devices. Here, we demonstrated that by replacing the original X-type ligand (R-COO^-^) with triethylborate (TEB), zinc-blend structure nanoplatelets (Zb-NPLs) turn from hydrophobic to hydrophilic and are quite stable in polar solvents. More importantly, a large shift of 253 meV is observed for the TEB-passivated NPLs, which can be attributed to the strain of the crystal lattice and the electron or hole delocalizing into the ligand shell. It is worth noting that unlike conventional inorganic ligands, such as metal chalcogenide complexes or halides that quench fluorescence, TEB-treated NPLs maintain 100% of their original brightness in polar solvents with a slight increase in full width at half maximum (FWHM, 32 nm). Furthermore, we explored the possibility of employing TEB as surface ligands for NPLs with different thicknesses and compositions. We believe the discovery of new surface chemistry using borate-related ligands can greatly expand the potential application areas of NPLs.

## Introduction

Two-dimensional semiconductor materials fascinate researchers with their outstanding electrical, and optical properties (Cai et al., 2018; Dong et al., 2018; Li et al., 2018; Wang et al., 2018; Zeng et al., 2018; Meng et al., 2019). In 2006, Heyon’s group reported wurtzite-type (Wz-type) two-dimensional CdSe NCs (Joo et al., 2006). Later, Zb CdSe NPLs were synthesized and reported in 2008 (Ithurria and Dubertret, 2008). Compared with traditional continuous growth, NPLs exhibit “discrete growth” and possess atomic-scale precision in the thickness direction (Riedinger et al., 2017; Pun et al., 2021). Therefore, the optical properties are closely related to the thickness. The emission spectra of NPLs are not continuous and show discrete colors including blue, green, and red, with the change in the number of layers (Porotnikov and Zamkov, 2020). Furthermore, the uniform thickness with atomic precision determines the narrower PL spectrum of NPL compared to other NCs (Porotnikov and Zamkov, 2020). Therefore, NPLs exhibit incomparable advantages over other NCs in many fields due to their specific optical and electrical properties (Fan et al., 2015; Zhang et al., 2016; Li et al., 2018; Guzelturk et al., 2019; Vanorman et al., 2020).

Apart from intrinsic features, ligands also have important effects on the properties of nanocrystals (Chaves et al., 2009; Lee et al., 2011; Boles et al., 2016; Scalise et al., 2018). The exciton radius of quantum dots can be tuned by certain ligands, which leads to a significant energy shift in the spectrum of NCs. For example, phenyldithiocarbamate (PTC) ligands caused the delocalization of holes and reduced the optical bandgap of CdSe NCs by 0.2 eV (Frederick and Weiss, 2010). The spectra of Wz-CdSe NPLs underwent a large redshift when the original L-type ligands were replaced by Z-type ligands which was attributed to changes in surface strain and effective thickness after ligand exchange occurred (Zhou et al., 2015). The photoluminescence quantum yield (PLQY) of Zb-CdSe NPLs in nonpolar solvents could be improved by replacing the native ligands with halogens and L-type ligands (Dufour et al., 2019).

As seen from the above examples, rationally designing ligands to modulate the properties of NPLs is important in future studies. It is well known that the properties of NCs in polar phases are important as this may affect the effectiveness of energy storage and conversion. (Naina et al., 2021), biomedicine (Gao et al., 2021), etc. However, research on the fluorescence properties of NPLs in polar solvents is extremely limited, because they are not stable in polar solvents due to aggregation, etching, and digestion (Sun and Buhro, 2020). In addition, the fluorescence quenching of NPLs in polar solvents is also an obstacle that needs to be overcome at present (Diroll and Schaller, 2019).

Here, we found that TEB can replace the natural ligands on the surface of Ⅱ-Ⅵ NPLs (CdS, CdSe). More importantly, we found that the absorption and emission of Zb-CdSe NPLs can be tuned by the surface environment. A large shift of 253 meV is observed for the borohydride-passivated sample, which is attributed to the strain of the crystal lattice and the electron or hole delocalizing into the ligand shell. It is worth noting that unlike conventional inorganic ligands, such as metal chalcogenide complexes or halides that quench fluorescence, TEB-treated NPLs maintain 100% of their original brightness in polar solvents with a slight increase in full width at half maximum (FWHM, 32 nm). Furthermore, we explored the possibility of employing triethylborate as a surface ligand for NPLs with different thicknesses and compositions. We believe that the discovery of new surface chemistry by introducing borate-related ligands can enrich the fundamentals and applications of NPLs.

## Materials and Methods

### Chemicals

Cdmium acetate dihydrate (Cd(Ac)_2_·2H_2_O, AR), lithium triethylborohydride (1.0 M solution in THF) were purchased from Shanghai Macklin Biochemical Co., Ltd. Myristic acid (AR, ≥99.0%), sodium hydroxide (NaOH, AR, ≥96%), N, N-dimethylformamide (DMF, AR, ≥99.5%), anhydrous ethanol and methanol (EtOH and MeOH, AR, ≥99.5%) were obtained from Sinopharm Chemical Reagent Co., Ltd. Oleic acid (OA, 90%) was purchased from Alfa Aesar. Selenium powder (Se, ≥99.99% metals basis,≥200 mesh), and octadecene (ODE, GC, >90.0%) were purchased from Aladdin. All chemicals were used as received without further purification.

### Preparation of Nanoplatelets

The synthesis of cadmium myristate was performed according a previous report with slight modification (Ithurria et al., 2011b): 0.24 g NaOH and 1.37 g MA were fully dissolved in 240 ml MeOH with 1 h of vigorous stirring. Then, 0.617 g of Cd(NO_3_)_2_·2H_2_O was dissolved in 40 ml of MeOH and slowly added to the above solution. Subsequently, a white precipitate was obtained. The resulting precipitate was rinsed three times with MeOH under vacuum filtration. Finally, it was transferred to a vacuum drying oven at room temperature overnight. The resulting white product is Cd(myristate)_2_.

NPLs were synthesized according to the previous literature (Ithurria et al., 2011a) (She et al., 2015). For 3.5 ML NPLs, 240 mg of Cd(Ac)_2_·2H_2_O, 150 µL of OA and 15 ml of ODE were degassed in a three-necked flask for one hour at 80°C. Then we raised the temperature to 180 °C and quickly injected 150 µL of TOP-Se (1 M). After washing three times with toluene, the products were dispersed in toluene. For 4.5 ML NPLs, 170 mg Cd(myristate)_2_ and 12 mg selenium powder were put into a three-neck bottle with 15 ml ODE as the solvent and degassed at room temperature for one hour. The reaction temperature was set to 240°C, where 40 mg of Cd(Ac)_2_·2H_2_O was quickly added when the temperature was raised to 190°C. After reacting at 240°C for 5 min, the three-neck bottle was quickly cooled to room temperature and 2 ml OA and 10 ml hexane were injected at 80°C. The precipitate containing NPLs after centrifugation of the mixture was redispersed in toluene. For 5.5 ML NPLs, in a three-neck flask, 170 mg of Cd(myristate)_2_ and 14 ml of ODE were degassed for 30 min at room temperature. Then, under nitrogen flow, the flask was heated to 240°C and 1 ml of a 0.15 M solution of selenium powder sonicated in ODE was injected. After 20 s, 60 mg of Cd(Ac)_2_·2H_2_O was introduced. The solution was held at 240°C for 10 min, and the temperature was then rapidly reduced to stop the reaction. A mixture solution containing 2 ml of OA and 15 ml of hexane was added. The mixture was then centrifuged, and the precipitate containing the NPLs was resuspended in toluene.

### Ligand Exchange

A two-phase method was used for ligand exchange in this experiment. The detailed process is as follows: 100 μL of DMF was added dropwise to a total volume of 1.6 ml of superhydride solution with a concentration of 1 M (in THF), and the reaction was carried out under magnetic stirring for 24 h in a glove box at room temperature. Subsequently, the sample was removed from the glove box and dried at room temperature by vacuuming. Finally, a transparent gel-like substance was obtained as the final product, triethylborate (TEB).

In a typical ligand exchange process, 1 ml of NPL solution (0.2 mg/ml) in hexane was mixed with 1 ml of DMF containing 2.5 mg of TEB. Under vigorous magnetic stirring overnight, the nanoplatelets transferred from the nonpolar phase to the polar phase. The bottom phase was separated and rinsed with fresh toluene one time to remove organics. The sample was also washed with DMF three times to remove excess TEB. Afterward, the TEB-capped NPLs were redispersed in DMF. Before ligand exchange, the NPLs were named 3.5 ML-OOCR, 4.5 ML-OOCR and 5.5 ML-OOCR. The samples after ligand exchange were named 3.5 ML-TEB, 4.5 ML-TEB and 5.5 ML-TEB.

## Characterization

UV-Vis absorption spectroscopy of NPLs was recorded by an Agilent Cary 5000 Spectrophotometer. The PL curves and decay were obtained on a HORIBA FL-3 3D Fluorescence Spectrometer. Fourier-transform infrared (FT-IR) spectra were acquired in transmission mode or ATR mode using a Nicolet iS50 FTIR spectrometer. The structure of samples was characterized by a Bruker D8 X-ray Powder Diffractometer, which was operated at 40 mA and 40 kV. Zeta potential was measured with a Malvern Nano-Z Zeta-Potential Analyzer. A JEOL JEM-2800 transmission electron microscope (TEM) was used for the TEM investigations of different samples.

## Results

The NPL studies in this work were synthesized following previously reported procedures using organic ligands such as OA and MA and dispersion in hexane (see Experimental Section) (She et al., 2015). The TEB was obtained in a glove box by mixing DMF with superhydride in THF and the corresponding reaction is as follows (Brown and Kim, 1977):

Superhydride itself can be considered a hole sacrificial agent and can quench the original fluorescence of the NCs when it is added to the NC solution directly (Tsui et al., 2016; Wang et al., 2021). However, in our study, the reducibility of superhydride was largely reduced, resulting in TEB functioning as a new type of ligand to stabilize NPLs in polar solvents. In the following sections, we will summarize and discuss the results obtained from NPLs stabilized by TEB ligand.

**Surface Engineering of Zb-NPLs.** To exchange the organic ligands, a two-phase surface treatment approach was used. The NPL dispersion was loaded on top of the ligand solution. Since the polarity of the solvents in the NPL dispersion and ligand was different, two phases were formed and the ligand exchange reaction involved a phase transfer process.

**FIGURE 1:**
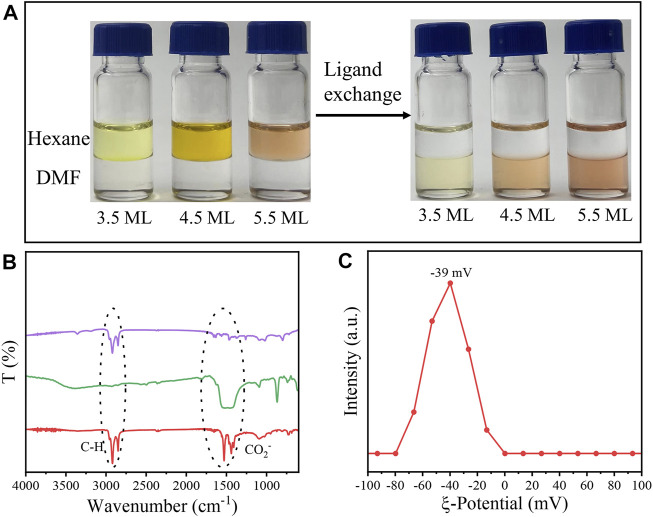
**(A)** Different monolayer CdSe NPLs undergo phase transfer from hexane to DMF. **(B)** FT-IR spectra (red: original NPLs, green: NPLs capped with TEB, purple: supernatant after ligand exchange). **(C)** Zeta potential for NPLs capped with TEB.

As shown in Figure 1A, Zb-CdSe NPLs with a thickness of 3.5, 4.5 and 5.5 ML were stabilized in DMF after ligand exchange, forming colloidal solutions. Interestingly, a color change of the NPL solution was observed, for example the 4.5 ML NPLs turned from yellow–brown to orange-red. The surface chemistry information of NPLs was explored by Flourier transform infrared (FT-IR) spectroscopy (Figure 1B). The stretching vibration at 2900 cm^−1^ belonging to C-H, and the symmetrical and asymmetric stretching vibrations at 1529 and 1435 cm^−1^ corresponding to -COO^-^ almost completely disappeared after ligand exchange (Figure 1B). Instead, TEB capped CdSe NPLs showed a broad peak centered at 1440, which was attributed to the stretching vibration of the B-O bond (Gautam et al., 2012). The absorption at 1089 cm^−1^ can be attributed to the vibration of the C-B bond according to the literature reports (Spitler and Dichtel, 2010; Wang et al., 2015). The supernatant after ligand exchange was also characterized by FT-IR. As seen in Figure 1B (purple curve), FT-IR showed strong C-H absorption (purple line), which further confirmed the efficacy of TEB ligands in complete removal of the original ligands of the NPLs. The negative *ζ* potential of -39 mV shown in Figure 1C proved that the NPL surface was negatively charged after the exchange. Such a negative value was caused by the TEB ions bound to the NPL surface, forming an electrical double layer around each NPL and was sufficient for electrostatic stabilization of the colloidal dispersion. The composition of the NPLs before and after treatment was analyzed and characterized by ICP. The results shown in Supplementary Table S1 confirmed the presence of boron ions on treated NPLs and indicated TEB as X type ligands bound to NPLs. We then explored the binding ability of this type of ligand to NPLs. NPLs capped with TEP were exchanged back to the nonpolar phase with OA and Olam, and the surface chemical information of the nanocrystals was characterized by FT-IR after thorough cleaning to remove excess organic matter (Supplementary Figure S1). The C-H (2900 cm^−1^) and -COO^-^ (1529 and 1435 cm^−1^) vibration signals of OA capped NPLs were obvious, while the C-B bond vibration signals located at 1440 cm^−1^ disappeared. Interestingly, the sample after exchange with Olam still had an obvious C-H vibration signal, and the C-B vibration did not disappear. We speculate that unlike OA ligands, amine-type ligands may link to this new ligand by hydrogen bonding.

**FIGURE 2:**
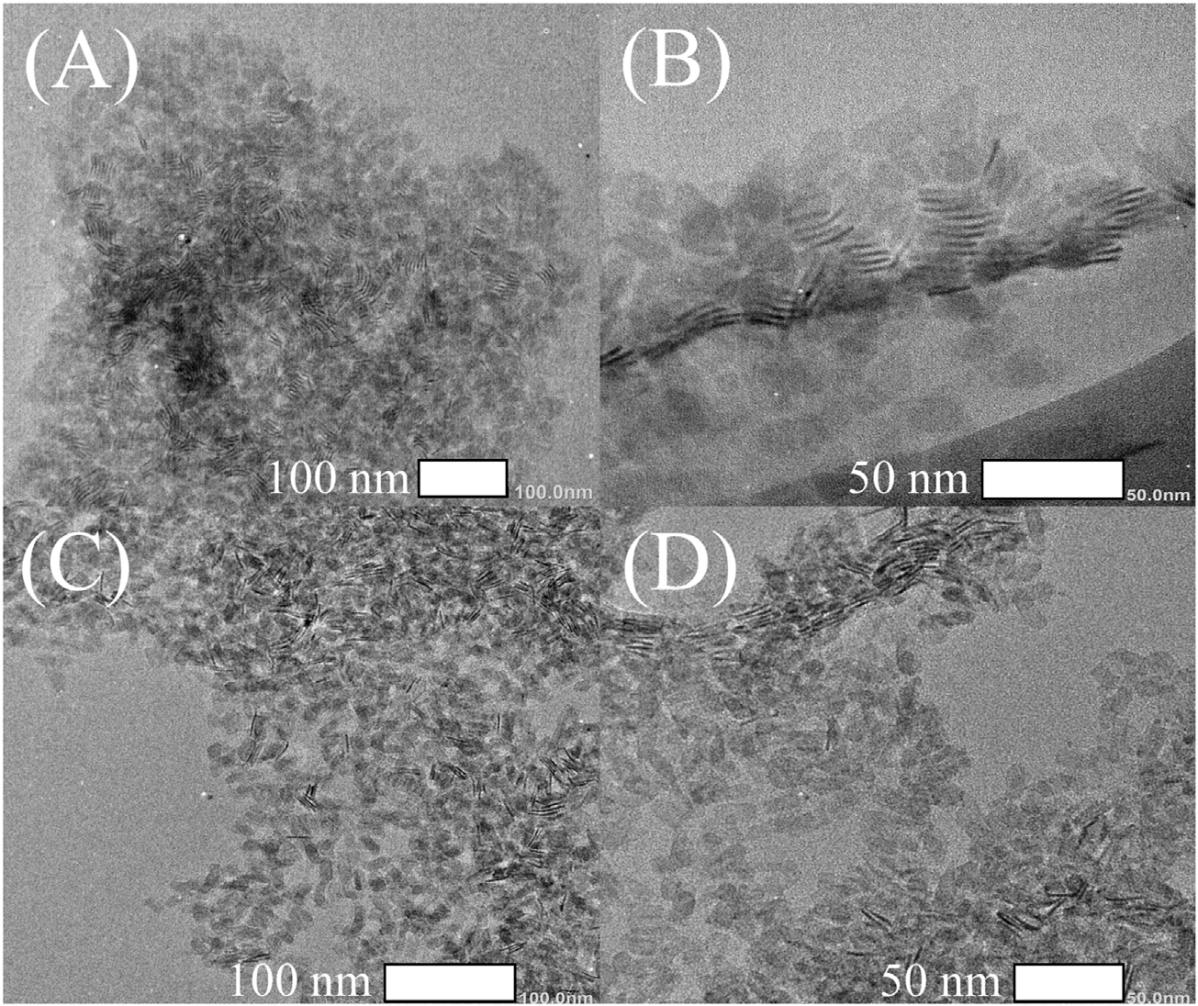
TEM images of 4.5 ML CdSe NPLs capped with different ligands **(A,B)** RCOO-and **(C,D)** TEB.

**FIGURE 3:**
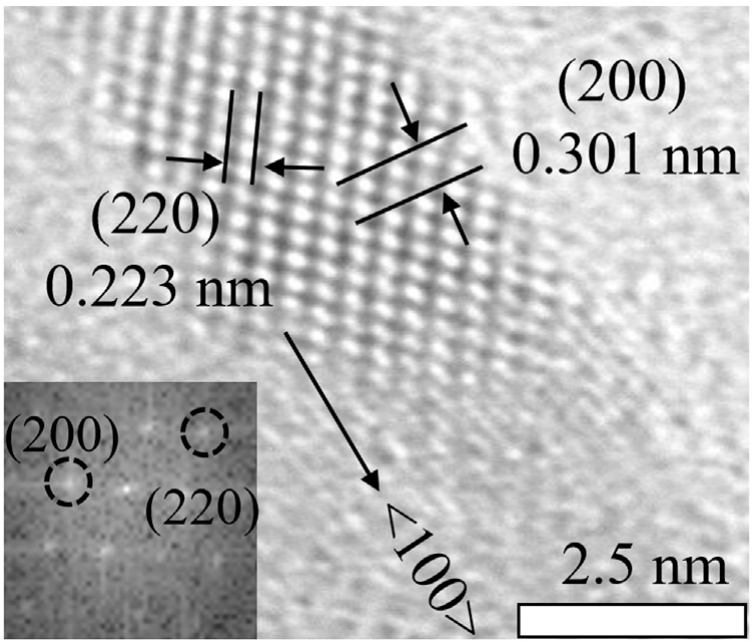
High resolution TEM image of the crystal lattice and the corresponding FFT patterns.

Subsequently, the morphology of the NPLs was obtained by TEM. Figures 2A,B shows that the size of the original NPLs was approximately 10 × 25 nm^2^. Importantly, the morphology and size of NPLs did not change significantly during the ligand exchange process (Figures 2C,D). The lattice parameters of NPLs were also measured by high-resolution TEM, and the result is shown in Figure 3. The lattice spacings of 0.301 and 0.223 nm belonged to the (200) and (220) crystal planes of NPLs, respectively. This value was lower than that reported in the literature (Yoon et al., 2021) because the surface strain of the nanocrystal changed after ligand exchange. The surface strain is one of the reasons for the changes in the optical properties of NPLs (Zhou et al., 2015) (Dufour et al., 2019). In addition, XRD data were also consistent with this phenomenon, supporting this speculation (see below for details).

TEM images of NPLs with different numbers of layers are also given, and the results are shown in Supplementary Figure S2. It can be seen from the figure that before the exchange, the size of the 3.5 ML NPLs was very large and curled together (Supplementary Figure S2A), and they were obviously loose after the exchange (Supplementary Figure S2B). This is because the change in the ligand releases the surface strain of the materials. Same phenomenon was observed in 5.5 ML NPLs as shown in (Supplementary Figures S2C,D). In this experiment, after ligand exchange, the ligand exchange did not destroy the original morphology and structure of NPLs and the morphology of NPLs was well maintained regardless of the side size, which is different from some previous reports (Sun and Buhro, 2020).

**FIGURE 4:**
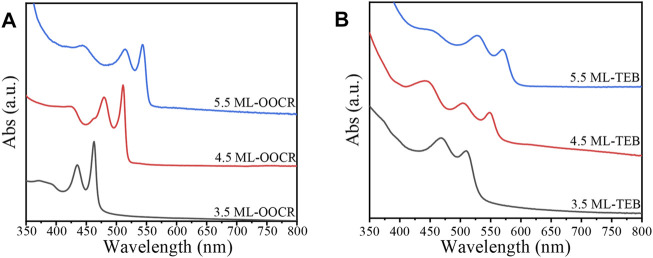
UV-vis absorption spectra of NPLs passivated with RCOO^−^ **(A)** and TEB **(B)**, respectively.

**The Optical Properties of NPLs.** Figure 4A shows the UV-vis absorption of the NPLs capped with organic ligands. For 3.5, 4.5 and 5.5 ML NPLs, the heavy holes located at 462, 511, and 543 nm respectively which were consistent with those of literature reports (Ithurria and Dubertret, 2008; Ithurria et al., 2011b). However, the HH (heavy hole), LH (light hole) and SO (spin orbit) of NPLs all underwent a large redshift after ligand exchange (as shown in Figure 4B). Among them, the HH of 3.5 ML was redshifted from the initial 462–510 nm, with a corresponding energy change of 253 meV, which was larger than that of 4.5 and 5.5 ML NPLs. Compared to the redshift generated by other ligands, the exciton energy shifts by TEB in this experiment were the largest. For example, Diroll and coworkers reported that the redshift of HH is 240 meV after treating 3.5 ML NPLs with halogen (Diroll and Schaller, 2019), and Zhou reported a large redshift up to 140 meV when wurtzite NPLs were treated with Z-type ligands (Zhou et al., 2015). We attributed the largest energy shift obtained in our system to the coeffect of surface strain and exciton delocalization on the NPLs. Details will be discussed in the Discussion section. In addition, comparing Figures 4A,B, it can be seen that the absorption peaks of NPLs are significantly broadened after ligand exchange, which is because the curvature of the valence band has changed with ligand exchange (Diroll, 2020).

Different from Wz-typle NPLs, the large exciton energy shifts on Zb-NPLs obtained by TEM treatment were partially reversible. Here, we used 4.5 ML NPLs as an example to further explore their optical properties, and the results are shown in Supplementary Figure S3. NPLs capped with TEB were exchanged back to the nonpolar phase *via* OA or Olam (Supplementary Figure S3). However, there were obvious differences between the two cases. The HH, LH and SO of the NPLs shifted to their original positions when exchanged back to the nonpolar phase with OA, similar to previous reports (Zhou and Buhro, 2017; Sun and Buhro, 2021), while Olam did not produce a similar effect. We speculated that Olam (L-type) combines with TEB through hydrogen bonds (Dufour et al., 2019). Therefore, the energy shift does not change. The above results further illustrated that the binding ability of ligands to nanocrystals decreases sequentially from oleic acid, TEB and Olam, which is confirmed by the results in Supplementary Figure S1.

**FIGURE 5:**
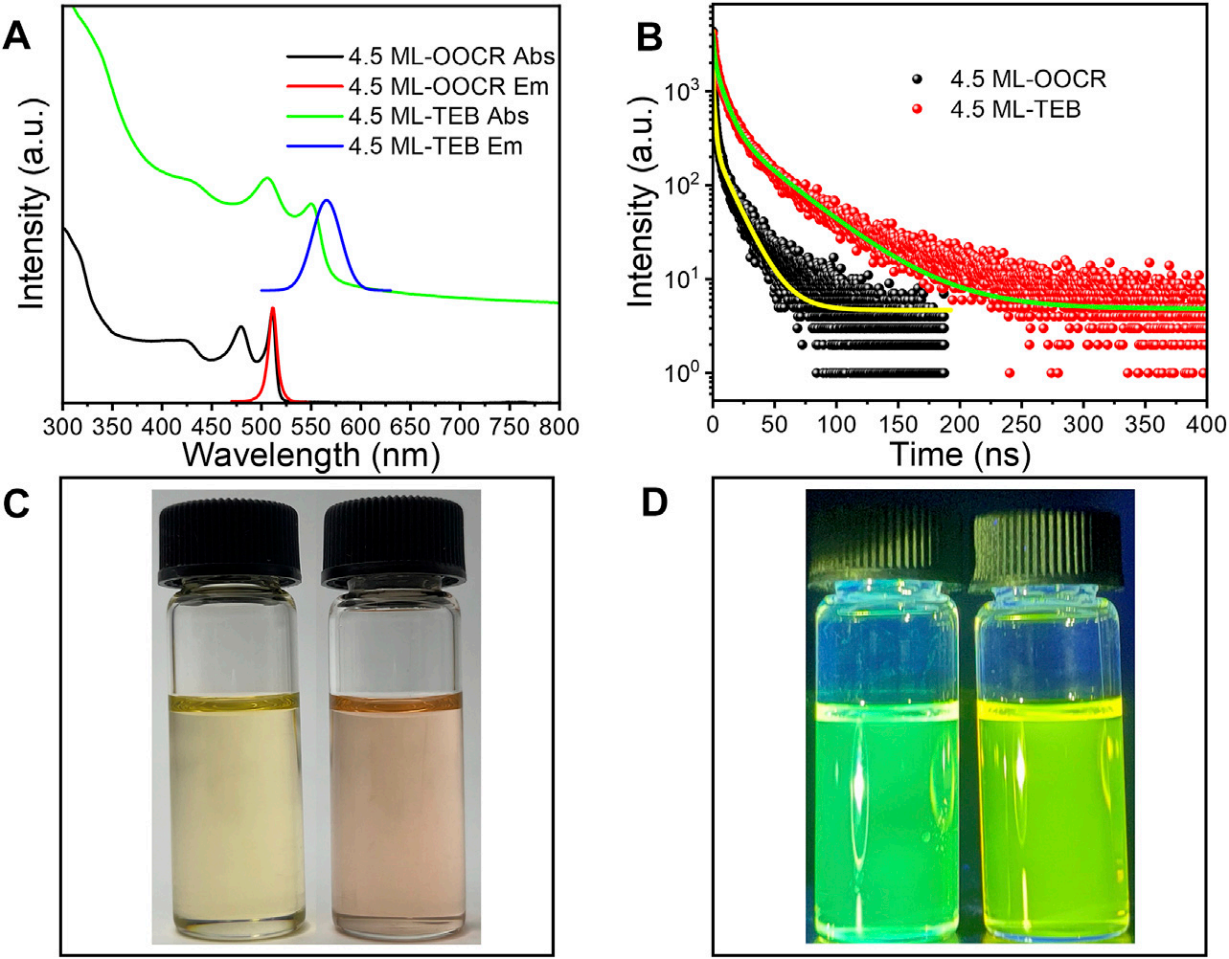
**(A)** UV-vis absorption and PL spectra of CdSe nanoplatelets capped with RCOO^−^ or TEB, respectively. **(B)** The PL decay curves of NPLs capped with RCOO^−^ and TEB. **(C,D)** The corresponding images of NPLs irradiated with white light and UV light (365 nm), respectively. **(C,D)** The CdSe NPLs capped with RCOO- (left) and TEB- (right) irradiated with white light and UV light (365 nm), respectively.

Interestingly, when NPLs were dispersed in DMF, they still preserved strong fluorescence, as shown in Figure 5A. Compared with NPLs capped with RCOO^−^, the HH of NPLs-TEB was redshifted to 550 nm, and the maximum fluorescence emission position was redshifted to 565 nm. The corresponding Stokes shift also increased. The PLQY of NPLs increased from 17.64 to 20.42% (Supplementary Figure S2). It is worth noting that the high fluorescence quantum efficiency of NPLs in polar solvents has not been reported before. The improved ligand coverage is a key factor in maintaining the original PLQY. The fluorescence lifetime was also measured, as shown in Figure 5B. The average fluorescence lifetime of natural NPLs was 9.76 ns, which increased to 17.11 ns after ligand exchange (Table 1). The reason why the fluorescence lifetime of NPLs capped with TEB is significantly increased compared to that of NPLs capped with RCOO^−^ is explained in the following section.

**TABLE 1:** The lifetime of 4.5 ML ZB CdSe NPLs capped with RCOO^−^ or TEB (ns).

Sample	A_1_	τ_1_	A_2_	τ_2_	A_3_	τ_3_	*τ*_avg_
4.5 ML-OOCR	0.711	0.1676	0.23	1.5834	0.06	14.5380	9.76
4.5 ML-TEB	0.615	0.6234	0.284	4.9300	0.101	26.01	17.11

**TEB as a Ligand for Other NPLs.** To fully illustrate the generality of this surface treatment method, TEB was used to treat other nanocrystals, such as CdS NPLs. CdS NPLs were synthesized according to the literature reports (Zhang et al., 2021), and the original ligands on the surface were MA. The ligand exchange procedure was conducted in the same manner as that of CdSeNPLs.

**FIGURE 6:**
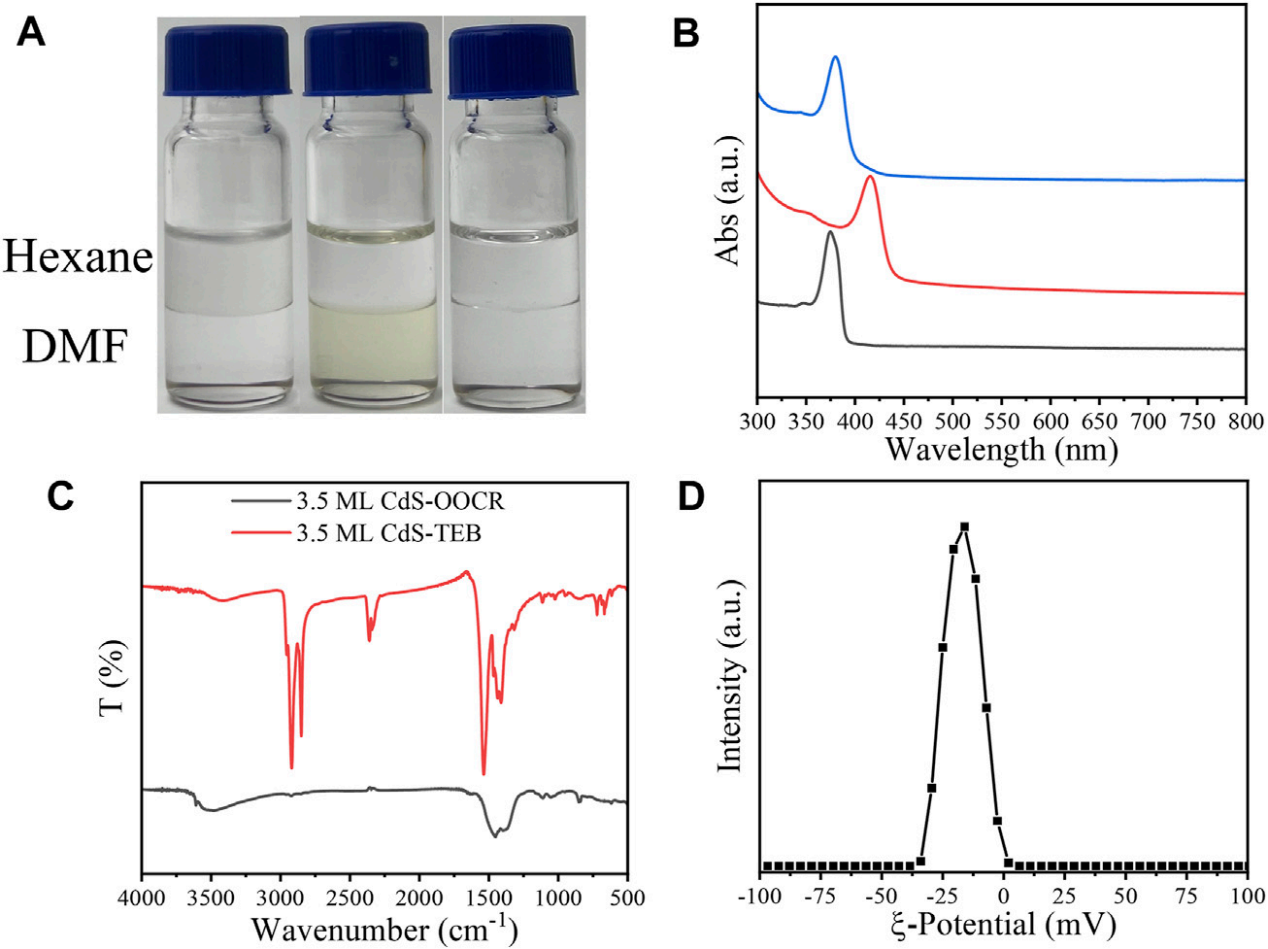
**(A)** 3.5 ML CdS NPLs undergoes phase transfer from hexane to DMF in the presence of TEB. **(B)** UV–vis absorption spectrum (black: original NPLs; red: NPLs capped with TEB; blue: back exchange with RCOO^−^), **(C)** FT-IR spectrum of 3.5 ML Zb-CdS capped with RCOO- and TEB. **(D)** The zeta potential of 3.5 ML ZB CdS-TEB in DMF.

After ligand exchange, CdS NPLs were transferred from hexane to the DMF phase. Similar to the CdSe NPLs, the color of the CdS NPLs also changed from white to pale yellow. The color of the CdS NPLs returned to white when exchanged back to the nonpolar phase with OA (Figure 6A). The HH absorption peak of the initial CdS NPLs is located at 374 nm, which was redshifted to 415 nm after the original ligand on the surface was replaced by TEB. The corresponding energy shift was 327 meV (Figure 6B). Exposure of any of the TEB capped NPLs to OA resulted in NPLs immediate back exchange to nonpolar phase. The back exchange from TEB to OA was evident by a return shift of the NPLs absorption features to the original position (Figure 6B, blue curve). The reversible shift on absorption spectra indicate that the CdS NPLs preserved their original 2D structures during surface treatment process, and further demonstrate the versatility of TEB ligand modification. In addition, a broadening of the absorption peak of the sample was also observed here which is similar to that of TEB for CdSe NPL. The surface chemical information of NPLs was recorded by FT-IR. As shown in Figure 6C, there was a strong absorption peak at 2900 cm^−1^, which corresponded to the C–H vibration of the original ligand. The two strong absorptions at 1530 cm^-^1 and 1420 cm^-^1 belonged to the symmetric and asymmetric stretching vibrations of -COO^-^ (Figure 6C, red line). The almost complete substitution of the original organic ligand was demonstrated by FT-IR after ligand exchange. From Figure 6C, it can be seen that the absorption signal of C-H is absent, and there is a strong broad peak near 1452 cm^−1^, which corresponds to the C-B bond. Finally, the *ζ* potential of the sample was -15 mV after ligand exchange (Figure 6D). This indicated that negatively charged TEB bound to the surface of NPLs and prevented the aggregation of NPLs. The above experimental results indicated that the new ligand, TEB, can also treat other NPLs in groups II-VI with the same effect.

## Discussion

**FIGURE 7:**
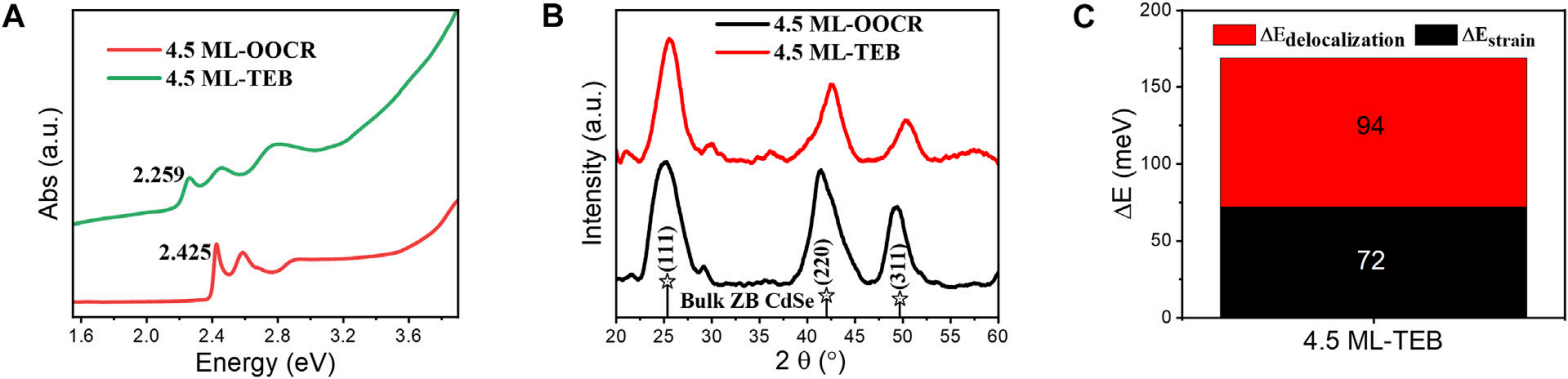
**(A)** UV-vis absorption spectra of CdSe nanoplatelets capped with RCOO^−^ or TEB. **(B)** High angle XRD data for CdSe nanoplatelets capped with RCOO^−^ or TEB of 4.5 ML. **(C)** Energy shift of the band gap for 4.5 ML NPLs.

**Mechanism of Large Exciton Energy Shift.** It is important to understand the changes in the optical properties of NPLs. To better explain the reason for the energy redshift of Zb-NPLs in this work, the structural information of the samples was characterized by XRD. As shown in Figure 7B, there were three obvious diffraction peaks for NPLs capped with RCOO^−,^ including 25.1°, 41.4° and 49.3°, which corresponded to the (111), (220) and (311) crystal planes of Zb-CdSe, respectively. After ligand exchange, NPLs retained obvious zincblende characteristics (Figure 7B). This showed that TEB did not destroy the crystal structure of NPLs. As shown in Supplementary Figure S4, the (220) crystal plane diffraction of NPLs split two diffraction peaks, including diffraction in the side and thickness directions. The lateral diffraction angle increased (red solid line), while the thickness direction diffraction angle decreased and widened (green solid line) after ligand exchange. The interplanar spacing was calculated from the Bragg equation, and the corresponding lattice parameters are given in Table 2. For example, the side distance is reduced from 2.187 to 2.118 Å. In contrast, the thickness direction was increased from 2.112 to 2.185 Å. Subsequently, the energy shift caused by the lattice strain change is calculated according to Equation 1. The HH of NPLs capped with organic ligands is located at 2.425 eV, but after TEB treatment, the HH of NPLs redshifted to 2.259 eV (ΔE = 166 meV). Therefore, the contribution of this factor to energy transfer was 72 meV, accounting for 43% (Figure 7C). Obviously, this value is less than half of the total energy shift.

**TABLE 2:** The lattice parameter information of NPLs for (220) (Å)

Sample	d_220, lateral_	d_220, thickness_	a	c	a/c
4.5 ML-OOCR	2.187	2.112	6.186	5.974	1.035
4.5 ML-TEB	2.118	2.185	5.991	6.180	0.969

The effect of exciton-delocalized ligands has been reported on dots, where the HOMO of the ligand is close to the VB of the NC so that the ligand can efficiently export photoexcited holes (Frederick and Weiss, 2010; Dufour et al., 2019). The NPLs used in our work represented classic examples of 2D structured NCs, in which the thickness was quantum confined. We proposed that excitation delocalization phenomenon could also be observed in 2D structure materials. When the NPLs capped with TEB were excited by rational light sources, the electrons delocalized to the conduction band, and the holes delocalized to the ligand shell. Due to the increase in the thickness of the quantum confinement, a large redshift occurs in both absorption and emission (Figure 5A). The lattice parameters and energy shifts of 3.5 and 5.5 ML NPLs are also given in Supplementary Table S3 and Supplementary Figures S7, S8 according to the XRD patterns. Similar to 4.5 ML NPLs, NPLs with 3.5 and 5.5 ML expanded in the thickness direction while compressed in the lateral dimension (Supplementary Figures S5, S6), corresponding to an increase in the *c* value and a decrease in the *a* value after ligand exchange (Supplementary Table S2). The contribution of exciton delocalization and surface strain to the energy shift for 3.5 and 5.5 ML was recorded in Supplementary Figure S8. It can be seen from the figure that the energy shift of 3.5 ML is the largest and 5.5 ML is the smallest. In addition, as the number of layers increased, the contribution of exciton delocalization to the energy shift gradually increased. Subsequently, the detailed fluorescence lifetimes of the samples were calculated according to the PL decay curve fitted with a third order exponential. From it can be seen that the proportion of A_1_ and A_2_ in NPLs before and after ligand exchange was not significantly different. However, A_3_ increased from the original 6–10.1% after ligand exchange. In addition, *τ*_3_ improved from 14.5380 to 26.01 s. The above results indicated the intervention of a nonradiative pathway related to the delocalization of electrons or holes into new ligand layers (McArthur et al., 2010; Kodaimati et al., 2018).

## Conclusions

In conclusion, a new ligand (TEB) was discovered in this experiment. This ligand can stabilize II-VI NPLs in DMF while maintaining the original morphology and crystal structure. Interestingly, TEB-passivated NPLs can retain 100% of the original brightness in polar solvents, which is beneficial for their application in devices. In addition, TEB can lead to a large redshift in the spectra of NPLs including absorption and emission. Further studies revealed that the two factors responsible for the redshift were changes in surface strain and exciton delocalization after ligand exchange. This means that we can tune the spectrum of NPL by ligands to suit the needs of the application. The development of borate-related ligands enriches the surface chemistry of Ⅱ-Ⅵ NPLs and introduces a practical pathway to the rational design of bright NCs for optoelectronic devices.

## Data Availability

The original contributions presented in the study are included in the article/Supplementary Material, further inquiries can be directed to the corresponding author.
