# Supplementary material for: Triethyl-Borates as Surfactants to Stabilize Semiconductor Nanoplatelets in Polar Solvents and to Tune Their Optical Properties
Source: Front Chem. 2022 Apr 12;10:860781. doi: 10.3389/fchem.2022.860781 (PMC9039045; doi:10.3389/fchem.2022.860781)
Supplement: Supplementary file 1 [file DataSheet1.pdf]

Supporting Information

Triethyl-borate as Surfactants to Stabilize Semiconductor Nanoplatelets in Polar Solvents and to Tune Their Optical Properties

Yalei Deng,[†] Xufeng Chen,[†] Jing Liang,[†] Yuanyuan Wang[†]*

[†] *State Key Laboratory of Coordination Chemistry, School of Chemistry and Chemical Engineering, Nanjing University, Nanjing 210093, China*

*To whom correspondence should be addressed. Email: wangyy@nju.edu.cn

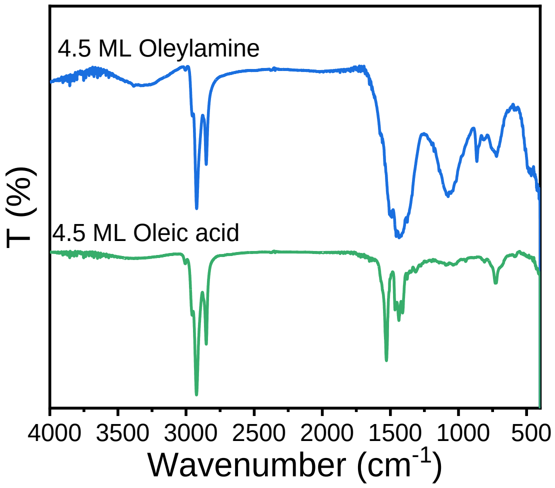

Figure S1| The FT-IR of 4.5 ML-TEB treated by OA or Olam back exchange in nonpolar solvent

Table S1 Element content of samples before and after TEB treatment (mmol)

	Cd	Se	B	Cd/Se
CdSe-OOCR	0.097	0.0746	0	1.30
CdSe-TEB	0.017	0.015	0.0165	1.13

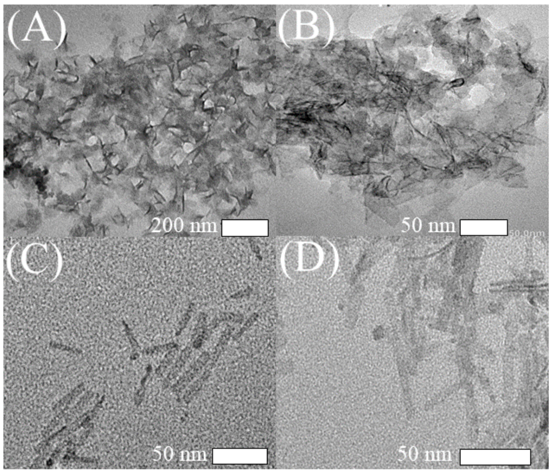

Figure S2| The TEM images of NPLs (a) 3.5 ML-OOCR, (B) 3.5 ML-TEB; (C) 5.5 ML-OOR, (D) 5.5 ML-TEB.

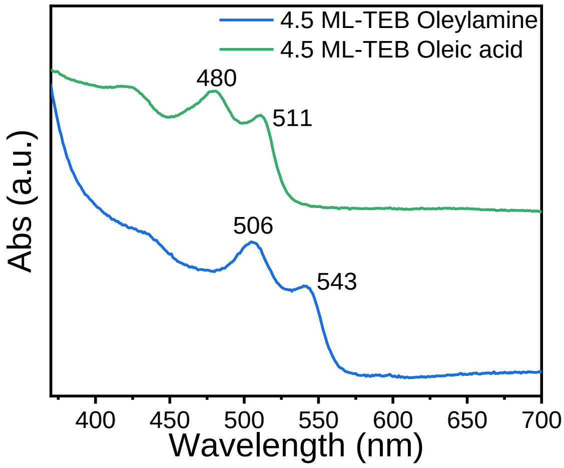

Figure S3| The UV-vis absorption of 4.5 ML-TEB treated by OA or Olam back exchange in nonpolar solvent

Table S2 Optical properties of NPLs capped with $RCOO^-$ and TEB

	HH (nm)	LH (nm)	SO (nm)	Em (nm)	PLQY (%)	Stock shift(nm)	FWHM (nm)
CdSe-OOCR	510	479	425	511	17.64	1	10
CdSe-TEB	550	506	431	565	20.42	15	32

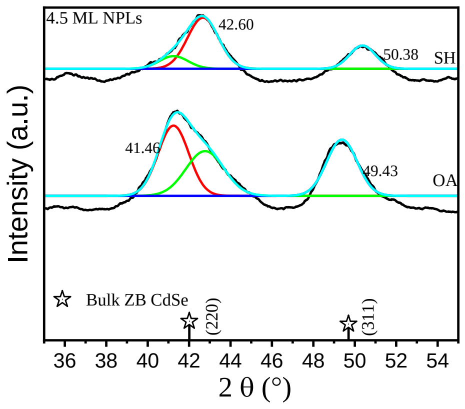

Figure S4| XRD pattern fitting curve of 4.5 ML NPLs.

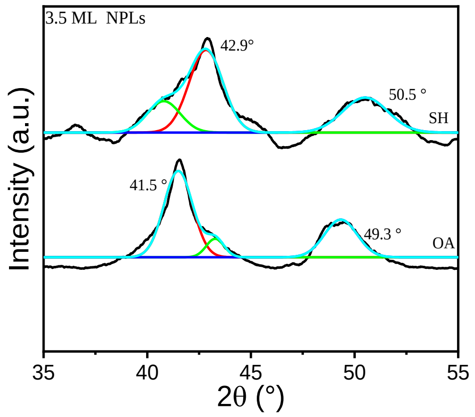

Figure S5| XRD pattern fitting curve of 3.5 ML NPLs.

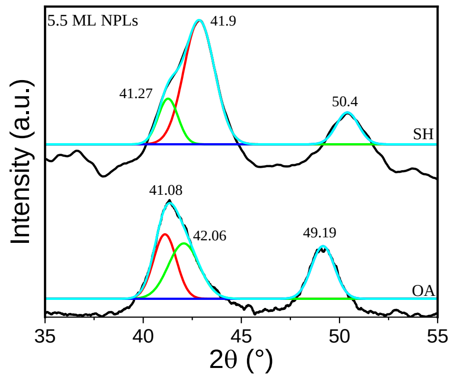

Figure S6| XRD pattern fitting curve of 5.5 ML NPLs.

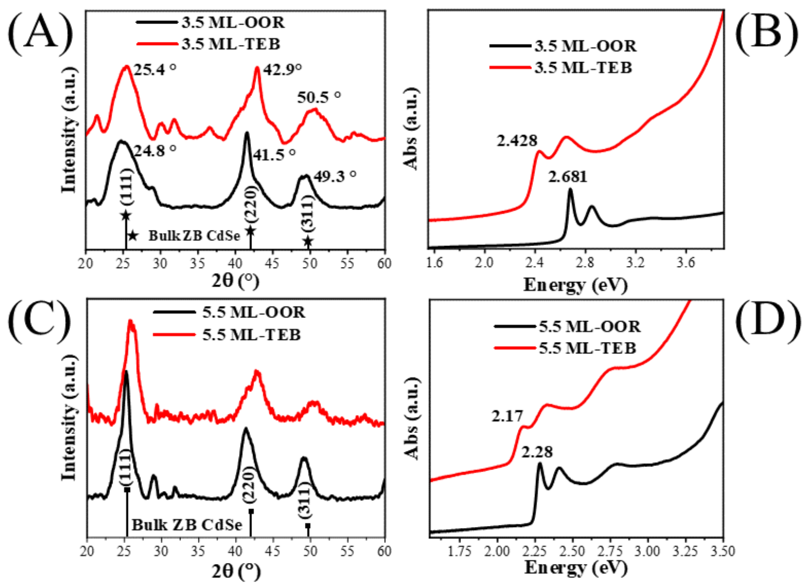

Figure S7| XRD pattern and UV-vis absorption curve of NPLs (A) and (B) 3.5 ML, (C) and (D) 5.5 ML.

Table S3. The lattice parameter information of NPLs for (220) (Å)

Sample	$d_{220,\ lateral}$	$d_{220,\ thickness}$	a	c	a/c
**3.5 ML-OOR**	**2.174**	**2.087**	**6.149**	**5.903**	**1.042**
**3.5 ML-TEB**	**2.109**	**2.209**	**5.965**	**6.248**	**0.955**
**5.5 ML-OOR**	**2.23**	**2.15**	**6.307**	**6.081**	**1.037**
**5.5 ML-TEB**	**2.11**	**2.19**	**5.968**	**6.194**	**0.963**

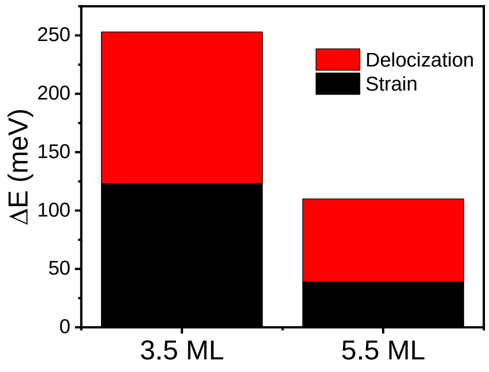

Figure S8| Energy shift of band gap for 3.5 and 5.5 ML NPLs.

Energy shift calculation(Zhou et al., 2015; Diroll and Schaller, 2019):

$$\Delta E = -\alpha \frac{C_X - C_{OA}}{C_{OA}} Y \quad (1)$$

α: Band gap pressure coefficient 43.1 meV/Gpa.

Y: Young's modulus 48.9 GPa.

Diroll, B. T., and Schaller, R. D. (2019). Shape-Selective Optical Transformations of CdSe Nanoplatelets Driven by Halide Ion Ligand Exchange. *Chem. Mater.* 31, 3556–3563. doi:10.1021/acs.chemmater.9b01261.

Zhou, Y., Wang, F., and Buhro, W. E. (2015). Large Exciton Energy Shifts by Reversible Surface Exchange in 2D II-VI Nanocrystals. *J. Am. Chem. Soc.* 137, 15198–15208. doi:10.1021/jacs.5b09343.
